# The Veterinary Profession’s Mecca in 1899

**Published:** 1899-07

**Authors:** 


### EDITORIAL.

### THE VETERINARY PROFESSION'S MECCA IN 1899.

All eyes are now turned toward the great metropolis of our land, and soon the tread of many footsteps will be wending their way to the scenes of our convention of 1899. It will meet for the first time under its new and broader name of the American Veterinary Medical Association, and no longer will there be a dividing line in thought or action at the borders of these United States. It is well that it holds its initial meeting under these broader auspices at its birthplace as a National Association, and where for more than a quarter of a century its strength of members was rallied from. It is pleasant to anticipate that there will be present on this auspicious occasion several of those who were numbered among the association's organizers, and whose allegiance to every progressive movement of the profession is to-day unsullied and unquestioned in devotion and untiring services for its welfare.

There will be many new faces, for it no longer gathers its strength from any sectional part of our land, but numbers its devotees in every state and territory, the Canadas and the great Northwest. In larger numbers than ever will they gather on this eventful occasion, and well may it be said, that they will come fully prepared and thoroughly imbued with the greater responsibilities of to-day, ready to discuss and consider the wider range of the veterinarian in the sanitary field, the laboratory, the surgical amphitheatre and the range of medicine, to give and receive with an unselfish spirit, that our whole people may reap the far-reaching benefits of the world of comparative medicine. Such a tribute is well deserved, such an honor is rightfully bestowed, and such a history worthily earned by the parent association that will step to the greater duties of the American Veterinary Medical Association.

